# *Take Five*: harmonization in personalized cancer vaccines for cancer immunotherapy

**DOI:** 10.1038/s12276-026-01807-y

**Published:** 2026-08-13

**Authors:** Seongje Cho, Jisun Lee, Young-Min Lee, Jae-Hwan Nam

**Affiliations:** 1 Department of Medical and Biological Sciences, The Catholic University of Korea, Bucheon, Gyeonggi-do Republic of Korea; 2 Department of Animal, Dairy and Veterinary Sciences, College of Agriculture and Natural Resources, Utah State University, Logan, UT USA; 3 BK Four Department of Biotechnology, The Catholic University of Korea, Gyeonggi-do Bucheon, Republic of Korea; 4 Korea National Institute of Health, Cheongju, Chungcheongbuk-do Republic of Korea

**Keywords:** Nucleic-acid therapeutics, Cancer immunotherapy

## Abstract

Precision medicine provides a therapeutic framework that addresses the interindividual diversity in tumor genetics, immunological determinants of disease, and immune responsiveness. Among precision approaches, personalized cancer vaccines (PCVs) have gained attention as a promising strategy for inducing tumor-specific immunity by identifying patient-derived neoantigens. Despite encouraging clinical outcomes, the development of PCVs faces major challenges in optimizing antigen selection, delivery, immune activation, and clinical translation. This Review summarizes current advances in PCV research, covering tumor antigen classification, neoantigen prediction algorithms, and the evolution of delivery technologies such as peptide, dendritic cell, DNA, and mRNA-lipid nanoparticle platforms. We highlight the advantages of mRNA-lipid nanoparticle systems that enable rapid manufacturing, potent antigen expression, and integration with immunostimulatory cytokines. Furthermore, we discuss emerging combination strategies involving cytokine engineering and T cell modulation that provide new opportunities to enhance immunogenicity and therapeutic efficacy. Ultimately, we propose that the future maturation of PCVs will require a coordinated process across five interconnected dimensions: (1) advances in neoantigen prediction technologies, (2) rapid delivery of PCVs to patients, (3) an increase in anticancer efficacy through combination therapy strategies, (4) establishment of cost-effective manufacturing processes, and (5) regulatory innovation. In this Review, we conceptualize this multidimensional alignment as “Take Five,” a unifying framework to bring precision, rhythm, and balance to next-generation cancer immunotherapy.

## Personalized cancer vaccines transform tumor immunity landscape

Personalized cancer vaccines (PCVs) represent a promising frontier in cancer immunotherapy, targeting patient-specific neoantigens to elicit robust immune responses. This Review introduces the “Take Five” framework, which outlines five strategic dimensions crucial for the successful development and clinical translation of PCVs: rapid treatment initiation, cost-effective manufacturing, regulatory innovation, enhanced anticancer efficacy through combination therapies, and advanced antigen prediction techniques. The authors discuss the integration of high-throughput sequencing, artificial intelligence-assisted prediction, and mRNA-lipid nanoparticle delivery platforms to overcome tumor heterogeneity and to induce durable antitumor immunity. Results from clinical trials in melanoma and pancreatic cancer demonstrate the potential of PCVs to generate significant immunological and clinical benefits. Future directions emphasize the need for synergistic optimization across these domains to transform PCVs into scalable, routine therapies, leveraging artificial intelligence-driven antigen discovery and innovative regulatory frameworks.

This summary was initially drafted using artificial intelligence, then revised and fact-checked by the author.

## Background of personalized cancer vaccines

Cancer involves profound genetic heterogeneity, with each tumor evolving as a distinct biological system shaped by the patient’s genome and environment^1,2^. This intrinsic diversity challenges conventional one-size-fits-all oncology approaches and underlies the rise of precision medicine, in which therapy is tailored to the molecular and immunological landscape of each patient^3,4^. The convergence of high-throughput sequencing, multi-omics analytics, and artificial intelligence (AI)-assisted prediction has enabled the resolution of the mutational architecture of individual tumors with unprecedented precision^5,6^. Within this precision framework, personalized cancer vaccines (PCVs) have emerged as transformative immunotherapies that target patient-specific neoantigens derived from somatic mutations to elicit potent cytotoxic T cell responses^7,8^. These neoantigens induce durable memory T cell responses and epitope spreading in patients with cancer^9–11^. By exploiting the mutational architecture of individual tumors, PCVs aim to convert the genetic instability of cancer cells into therapeutic vulnerability^12^.

**Fig. 1:**
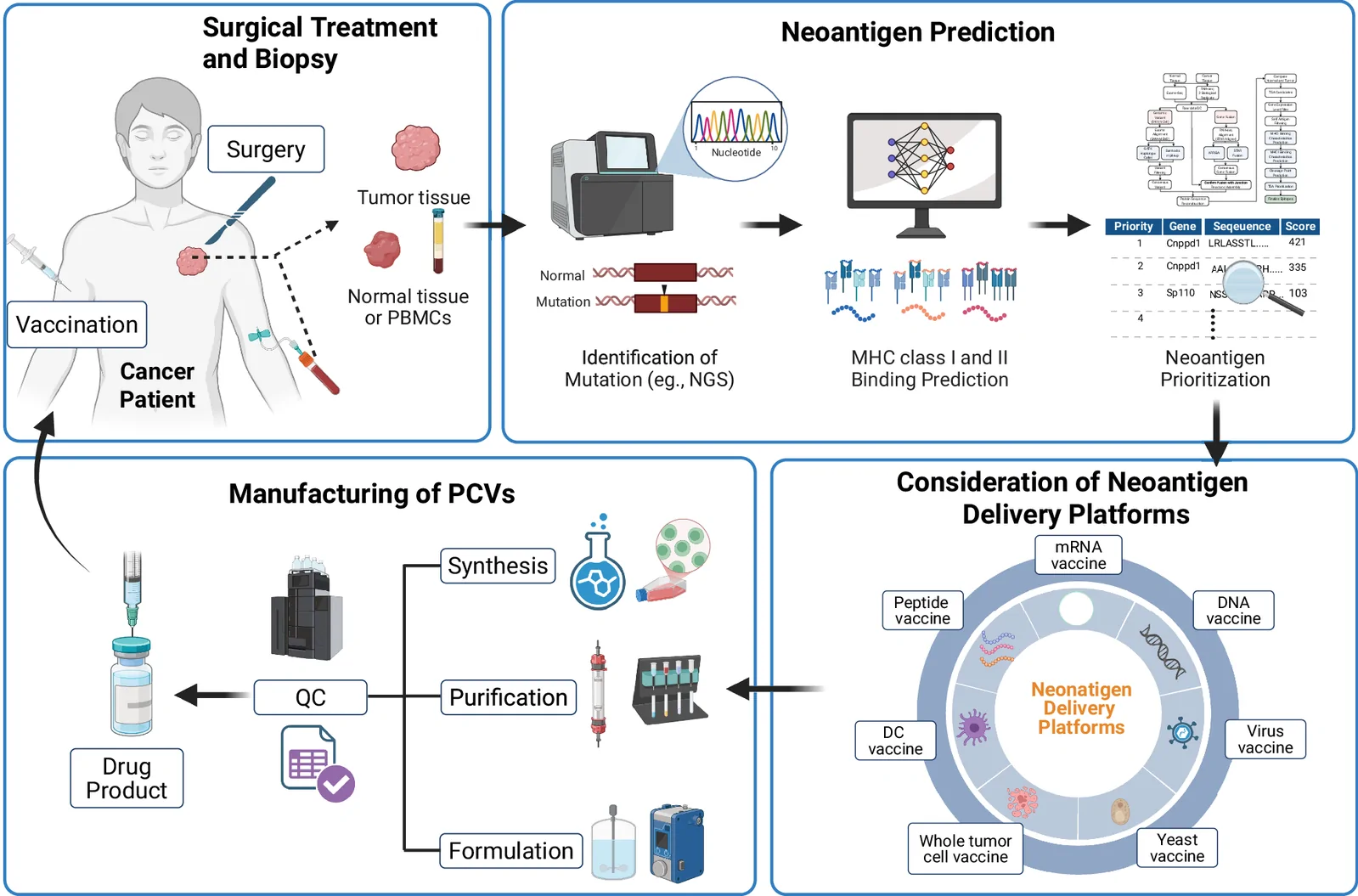
Process of PCV development. Personalized cancer vaccine (PCV) production begins with obtaining a biopsy of the patient’s tissue samples. To identify tumor-specific mutations, genomic information from the tumor tissue is compared with that from matched normal tissue using whole exome sequencing and RNA sequencing. Subsequently, neoantigen candidates are prioritized based on factors such as major histocompatibility complex (MHC) binding affinity and RNA expression levels. The predicted neoantigen candidates can be produced using various delivery modalities, each with its distinct advantages. PCVs are produced as individualized pharmaceuticals through synthesis, purification, and formulation processes to ensure their quality. Ultimately, PCVs aim to identify neoantigens from individual patients, manufacture them using an appropriate vaccine platform, and administer them back to the patients. DC, dendritic cell; NGS, next-generation sequencing; PBMC, peripheral blood mononuclear cell; QC, quality control.

As illustrated in Fig. 1, the PCV pipeline begins with the clinical acquisition of tumor and matched normal samples, followed by next-generation sequencing, and computational neoantigen prediction and prioritization^5,6^. Thereafter, high-value neoantigens are incorporated into diverse delivery platforms, including peptide, dendritic cell (DC), DNA, viral, and mRNA-lipid nanoparticle (mRNA-LNP) vaccines. Among these, mRNA-LNP vaccines offer rapid multi-epitope incorporation, efficient engagement of endogenous DCs, and robust T_H_1-skewed immunity without the use of external adjuvants. Vaccine synthesis, formulation, and stringent quality control ensure potency, sterility, and reproducibility. Together, these steps translate tumor-specific mutations into individualized immune responses capable of overcoming tumor heterogeneity and inducing durable antitumor immunity. However, mRNA-LNP-based PCVs face substantial translational challenges, including interpatient variability in innate RNA sensing, antigen processing, and T cell priming, as well as manufacturing bottlenecks and regulatory complexity^13^.

To address the challenges inherent in PCV development, we introduce the “Take Five” framework, a conceptual scaffold capturing five strategic dimensions required for successful development and clinical translation: (1) improvement of neoantigen prediction technologies; (2) rapid treatment initiation; (3) rational combination therapy; (4) cost-effective manufacturing systems; and (5) regulatory innovation. Together, these components place PCV efficacy at the intersection of computational precision, clinical timing, immunological synergy, scalable biomanufacturing, and adaptive regulatory science. In this review, we examined the scientific foundations and technological advances that drive mRNA-LNP-based PCVs. We discuss antigen classification, neoantigen discovery and prediction strategies, and the evolution of antigen delivery platforms. We further outline the mechanisms of immune activation and summarize the emerging clinical evidence. Finally, we discuss in detail the Take Five framework as a forward-looking guide for translating PCVs into routine clinical practice.

## Tumor antigens and neoantigen prediction

Selection of appropriate tumor antigens is a critical determinant of the efficacy of PCVs. Tumor antigens are broadly classified into tumor-associated antigens (TAAs), which are expressed in both normal and malignant tissues, and tumor-specific antigens, whose expression is restricted to cancer cells^14,15^. TAAs encompass overexpressed proteins, such as HER2, TERT, and survivin^16,17^; tissue differentiation antigens, such as PSA and MART1 (ref. ^18^); and cancer testis antigens, including the MAGE family and NY-ESO-1 (ref. ^19^). Although TAAs have been widely explored as immunotherapeutic targets, their expression in healthy tissues limits tumor specificity and raises concerns regarding off-tumor toxicity. By contrast, tumor-specific antigens, comprising oncogenic viral antigens and neoantigens derived from somatic mutations, are exclusively expressed in malignant cells and offer higher tumor specificity and potent immunogenicity^12,20–23^. Neoantigens, first described by De Plaen et al. in 1988 (ref. ^24^), represent the most promising substrates for PCVs because they bypass central immune tolerance and elicit robust T cell responses.

The development of PCVs requires the systematic identification and prioritization of patient-specific neoantigens. Tumors and matched normal tissues undergo next-generation sequencing (whole-exome, transcriptome, or RNA sequencing) to detect non-synonymous somatic mutations. Recently, advances in sequencing technology have substantially accelerated progress in neoantigen discovery. In particular, long-read sequencing technology has been shown to outperform conventional short-read sequencing in the detection of somatic structural variations in cancer genomes^25,26^. In addition to bulk sequencing approaches, emerging technologies such as single-cell RNA sequencing and spatial transcriptomics are providing deeper insights into the functional immunogenicity of neoantigens. These approaches enable simultaneous analysis of tumor cell transcriptional profiles and T cell receptor (TCR) repertoires at single-cell resolution, thereby allowing the identification of neoantigens that are actively recognized by T cells within the tumor microenvironment (TME). By linking antigen expression with immune cell reactivity, these methods help overcome the limitations of purely in silico prediction and improve the selection of neoantigens with a higher likelihood of eliciting effective T cell responses. Furthermore, spatial transcriptomics enables the investigation of spatial interactions between neoantigen-expressing tumor cells and infiltrating immune cells, providing important context for antigen presentation and T cell infiltration.

In parallel, liquid biopsy approaches based on circulating tumor DNA are emerging as minimally invasive tools for real-time monitoring of tumor evolution and dynamic changes in the neoantigen landscape. This strategy offers the potential to refine antigen selection over time and to correlate neoantigen dynamics with vaccine-induced immune responses, thereby supporting more precise evaluation and optimization of PCV strategies. Candidate neoantigens are further filtered based on their expression levels and predicted immunogenicity using major histocompatibility complex (MHC) class I and II binding algorithms, with subsequent in vitro validation to refine the final selection^11,27–30^. This approach enables the targeting of diverse mutation types, including point mutations and insertions/deletions, thereby maximizing the likelihood of eliciting effective anticancer immune response.

By integrating tumor antigen classification with computational neoantigen prediction, this framework ensures that PCVs are designed to achieve high tumor specificity, immunogenic potential, and clinical relevance. Despite these advances, the workflow for neoantigen selection remains heterogeneous across platforms and institutions, highlighting the need for standardized criteria and validation frameworks. Therefore, neoantigen-driven vaccine design represents one of the most rapidly evolving frontiers in personalized cancer immunotherapy. These considerations highlight the growing need for delivery platforms capable of faithfully presenting selected neoantigens and efficiently activating T cell responses.

Tumor mutational burden (TMB) is widely used as an indirect measure of neoantigen load and responsiveness to immune checkpoint blockade and, increasingly, to PCVs. Clinical experience with individualized mRNA-LNP vaccines for melanoma and pancreatic ductal adenocarcinoma (PDAC) illustrates the potential and limitations of this metric. In TMB-high tumors, such as melanoma, the abundance of somatic mutations increases the likelihood of generating immunogenic neoantigens and supports robust vaccine-primed T cell responses. By contrast, PDAC is typically a low-TMB, immunologically “cold” tumor; however, early-stage patients receiving resection followed by personalized vaccination can mount durable neoantigen-specific CD8⁺ T cell responses and achieve prolonged recurrence-free survival (RFS) in a subset of cases. These observations, together with broader correlative studies, indicate that bulk TMB alone incompletely captures the determinants of vaccine responsiveness; high TMB may be dominated by poorly immunogenic or subclonal mutations, whereas some low-TMB tumors harbor a small number of highly immunogenic, truncal neoantigens that are particularly attractive PCV targets. Consequently, TMB is now regarded as a coarse enrichment biomarker that must be integrated with information on the quality of neoantigens capable of effectively inducing cancer-specific immune responses, clonal architecture, antigen processing and presentation, and the TME, rather than a rigid eligibility threshold, for both neoantigen prediction pipelines and stratification of patients in PCV clinical trials^11,31,32^.

## Antigen delivery platforms

The clinical performance of PCVs depends on not only neoantigen selection but also the delivery modality used to present antigens to the immune system. Historically explored platforms include peptide vaccines, DC vaccines, and DNA vaccines, as well as more recent mRNA-LNP systems, each offering distinct biological and logistical attributes^1,33^ (Table 1).

**Table 1:** Comparison to neoantigen delivery modalities.

Category	mRNA vaccine	Peptide vaccine	DNA vaccine	DC vaccine
Antigen breadth	Multi-epitope, neoantigen-capable	Narrow, HLA-restricted	Moderate	Capable of utilizing diverse antigen forms
Production speed	Weeks	Days	Weeks	Weeks
Scalability	High	Moderate	High	Low
Cost considerations	Moderate	Low	Low	High
Immune response	Strong T cell and antibody	Weak (needs adjuvant)	Variable	High
Genomic safety	Non-integrating	Excellent	Potential insertion risk	Non-integrating
Clinical maturity	FDA-approved (BNT111, BTC); phase III ongoing (mRNA-4157)	FDA-approved (NeoVax, clinical trials ongoing)	Phase I–II (no approved products)	FDA-approved (Provenge, 2010)
Clinical response rates	ORR = 30.6%, CR = 8.3% (surgically resected PDAC), NCT04161755 (ref. ^70^), phase I	ORR = 59% (melanoma), 39% (NSCLC), and 27% (bladder cancer), NCT0897765 (ref. ^108^), phase Ib	ORR = 30.6% (advanced hepatocellular carcinoma), NCT04251117 (ref. ^109^), phase I/II	Objective effectiveness rate = 25% (refractory NSCLC), NCT02956551 (ref. ^110^), phase I
Recurrence reduction in clinical studies	44% reduction in recurrence (resected melanoma), NCT03897881 (ref. ^69^), phase IIb	Mean PFS = 3.1 months (advanced pancreatic cancer), NCT03645148 (ref. ^111^), phase I	36-month recurrence-free survival: 87.5% (TNBC), NCT02348320 (ref. ^112^), phase I	Three of six vaccinated patients experience disease recurrence during the follow-up period of 2 years (resected NSCLC), NCT0407826 (ref. ^113^), phase I
Survival trends in clinical studies	Median OS = 107.1 weeks (unresectable metastatic HPV − HNSCC), NCT03313778 (ref. ^114^), phase I	Mean OS = 24.1 months/8.3 months (non-squamous NSCLC), NCT03380871 (ref. ^115^), phase Ib	Median OS = 19.9 months (advanced hepatocellular carcinoma), NCT04251117 (ref. ^109^), phase I/II	Median OS = 7.9 months (refractory NSCLC), NCT02956551 (ref. ^110^), phase I
Limitation	Delivery dependency	Low immunogenicity	Low nuclear uptake	High cost

HLA, human leukocyte antigen; HNSCC, head and neck squamous cell carcinoma; HPV, human papillomavirus; NSCLC, non-small-cell lung cancer; PDAC, pancreatic ductal adenocarcinoma; PFS, progression-free survival; TNBC, triple-negative breast cancer.

Peptide vaccines were the earliest PCV platforms to leverage synthetic epitopes that mimic natural MHC-I (8–10 amino acids (aa)) and MHC-II (12–20 aa) antigen presentation^34^. Advances in solid-phase peptide synthesis have enabled scalable multi-epitope production^35^. However, their inherently low immunogenicity necessitates the use of strong adjuvants such as poly I:C^36,37^, and clinical responses have remained modest owing to limited cross-presentation, human leukocyte antigen (HLA) restriction, and vulnerability to tumor antigen heterogeneity.

DNA vaccines deliver plasmid constructs encoding tumor antigens and offer stability, ease of manufacturing, and the capacity to encode multiple epitopes within a single vector^38,39^. However, as plasmid DNA requires nuclear entry for transcription, in vivo antigen expression is inefficient and often necessitates electroporation or other physical delivery methods. Furthermore, the weak innate immunogenicity of DNA vaccines leads to suboptimal DC activation, and the theoretical risks of genomic integration limit their broader clinical adoption.

These challenges underscore the need for a platform capable of potent antigen expression, efficient engagement of endogenous DCs, and scalable personalization. mRNA vaccines overcome key mechanistic bottlenecks observed in peptide, DC, and DNA vaccines. They provide high-level antigen expression independent of HLA specificity, support efficient cross-presenting DC activation in vivo, elicit robust T_H_1-skewed immunity without external adjuvants, and are produced quickly with consistent manufacturing quality^40–45^.

## Mechanism of mRNA-based PCVs

**Fig. 2:**
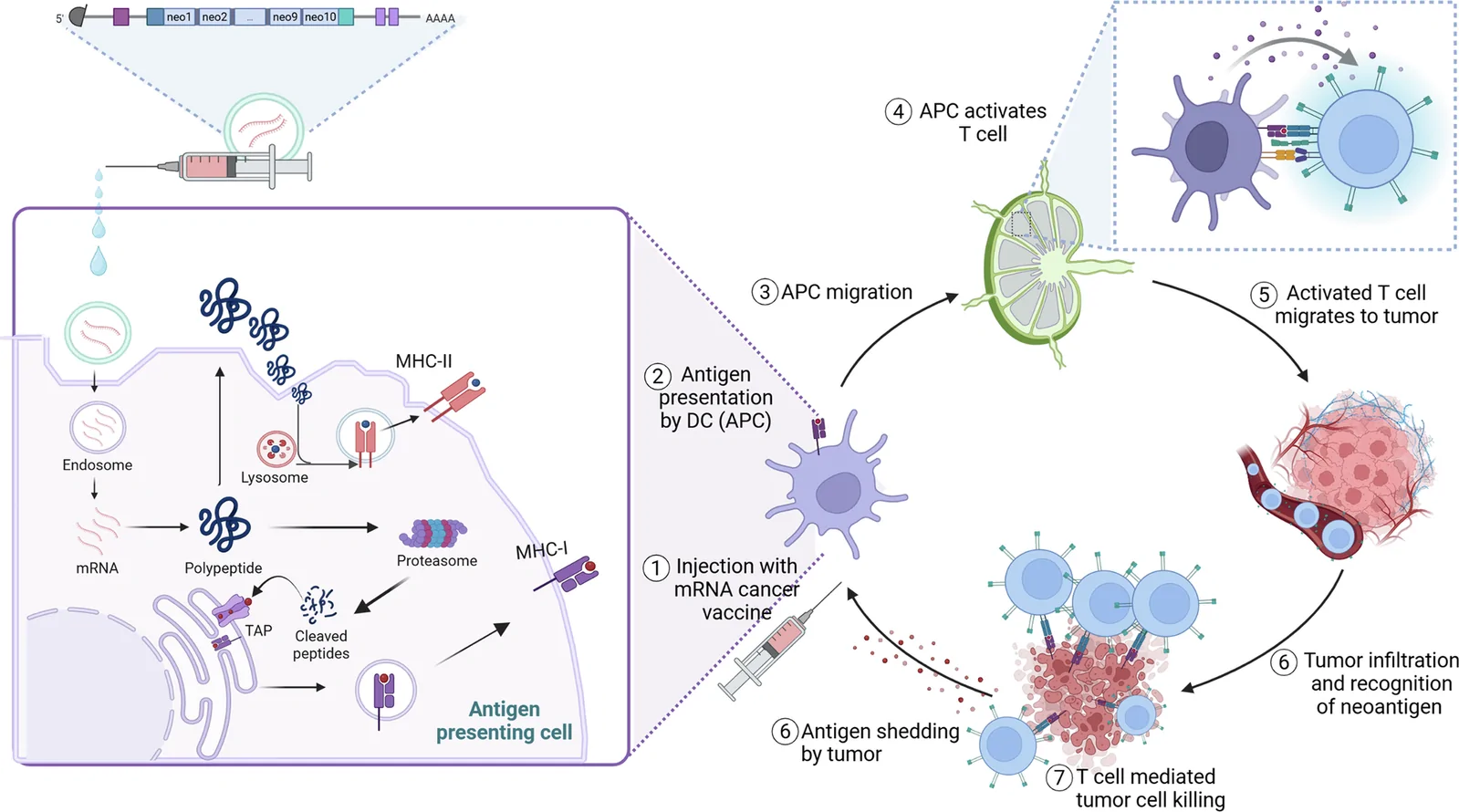
Mechanism of mRNA-based PCVs in vivo. Neoantigens encoding mRNA-lipid nanoparticles (LNPs) are endocytosed by antigen-presenting cells (APCs), such as dendritic cells (DCs), macrophages, monocytes, and B cells. mRNA is expressed as a protein that enters the cytoplasm via endosomal escape, and APCs present neoantigens through the major histocompatibility complex (MHC)-I or MHC-II pathways. APCs migrate into lymphoid organs and activate T cells. T cells infiltrate tumor tissues, recognize antigen-specific tumor cells, and kill them. PCV, personalized cancer vaccine; TAP, transporter associated with the antigen processing.

The mRNA-LNP platform is one of the most attractive delivery platforms for PCVs because of the following three advantages^46,47^: (1) the ability to deliver multiple epitopes with a single mRNA construct, (2) a simple and rapid manufacturing process independent of antigen sequences, and (3) potent induction of antigen-specific immune responses. mRNA-LNP platforms are well known for their mechanism of action through applications such as COVID-19 vaccines^42,48^. First, mRNA is processed within antigen-presenting cells (APCs), undergoes endosomal escape depending on endosomal pH, and is released into the cytoplasm to express peptides (Fig. 2). Peptides encoding antigen sequences are classified into two types based on their presentation pathways: MHC class I and MHC class II. MHC-I-restricted peptides are cleaved by the proteasome into adequate lengths of 8–13 aa, enabling them to form complexes with MHC-I molecules. Antigens with tumor mutations enter the endoplasmic reticulum via the transporter associated with the antigen processing (TAP) pathway, bind to MHC-I molecules, and are present on the cell surface. By contrast, MHC-II-restricted antigens, which typically have 13–25 aa residues, are acquired either through processing of extracellularly secreted peptides by lysosomes or via the cross-presentation pathway. APCs that present neoantigens with MHC molecules migrate to secondary lymphoid organs, including the lymph nodes and spleen. They present a neoantigen to naive T cells, and three signals have a key role in the activation of T cells: (1) the interaction between the antigen–MHC complex on APCs and the TCR on T cells; (2) the interaction of co-stimulatory molecules, such as the interaction between CD80/CD86 on APCs and CD28 on T cells; and (3) the signals of pro-inflammatory cytokines, such as IL-2 and IL-12, which are associated with the differentiation, proliferation, and survival of T cells. Activated T cells then migrate to peripheral tissues, including tumors, by expressing diverse chemokines. The tumor-infiltrating T cells activated by APCs recognize the MHC–neoantigen complex on the surface of tumor cells via their TCR and secrete pro-inflammatory cytokines and cytotoxic molecules such as interferon-gamma (IFNγ), tumor necrosis factor-alpha (TNF-α), perforin, and granzyme B. This process, mediated by antigen-specific T cell responses, not only directly kills cancer cells but also continuously induces antigen-specific immune responses and the release of cancer antigens from dead cancer cells.

Beyond direct tumor cell killing, mRNA-based PCVs can influence tumor progression more broadly by reshaping the metastatic potential of cancer cells and remodeling the TME. Vaccine-primed T cells circulate systemically and recognize shared neoantigens across distant or emerging metastatic sites, thereby contributing to the control of minimal residual disease and reducing the likelihood of metastatic outgrowth. Moreover, the influx of highly activated T_H_1 and cytotoxic T cells into the tumor bed drives the conversion of immunologically cold tumors into inflamed tumors, which are characterized by enhanced antigen presentation, increased chemokine production, and improved immune infiltration. T cell-mediated inflammatory reprogramming can suppress immunosuppressive components of the TME, such as regulatory T cells, myeloid-derived suppressor cells (MDSCs), and M2-like macrophages, while promoting conditions that favor durable anticancer immunity.

Nevertheless, applying the mRNA-LNP platform to PCV still presents challenges that need to be addressed. First, although mRNA manufacturing processes have been standardized and widespread following the COVID-19 pandemic, there is still a shortage of cGMP facilities capable of actual production^49^. Furthermore, given the nature of PCV, cGMP facilities capable of small-scale production — unlike those for preventive vaccines — may create a bottleneck in the clinical application of the mRNA-LNP platform^50^. Second, the mRNA-LNP formulations currently used in clinical trials must still be stored at temperatures between −20 °C and −80 °C, requiring a strict cold chain, which may pose logistical challenges^51^. Third, the self-adjuvant properties of mRNA-LNP formulations can induce a strong innate immune response to maximize anticancer effects^45,52^; however, this excessive innate immune response may increase side effects and suppress antigen expression, thereby inhibiting adaptive immune priming. Furthermore, considerable interindividual variability exists in innate RNA-sensing capacity, endosomal escape efficiency, and antigen processing, which collectively contributes to heterogeneous immunological outcomes across patients receiving otherwise identical mRNA-LNP constructs^45^.

In summary, despite these issues, the diverse mechanistic cascades triggered by mRNA-LNP vaccination position this platform as a highly promising approach for PCV development, as it enables precise neoantigen recognition while exerting broad immunological effects on metastasis suppression and the overall TME.

## T cell immunity

Effective PCVs rely on the coordinated activation of both CD8⁺ cytotoxic T cells and CD4⁺ T_H_ cells, which together shape the magnitude, quality, and durability of antitumor immunity. Following antigen presentation via MHC-I and MHC-II pathways, vaccine-primed CD8⁺ T cells recognize neoantigen-bearing tumor cells and execute cytolysis through perforin and granzyme B, while simultaneously producing IFNγ and TNF-α to remodel the TME and amplify downstream immunity^53–55^. Although multiple CD8⁺ T cell subsets such as Tc1, Tc2, and regulatory CD8⁺ T cells have been described, cytotoxic Tc1 cells remain the principal effectors of PCV-induced tumor control^56–59^. Sustaining their activity requires minimizing exhaustion, enhancing tumor infiltration, and limiting the number of suppressive cells within the TME.

**Fig. 3:**
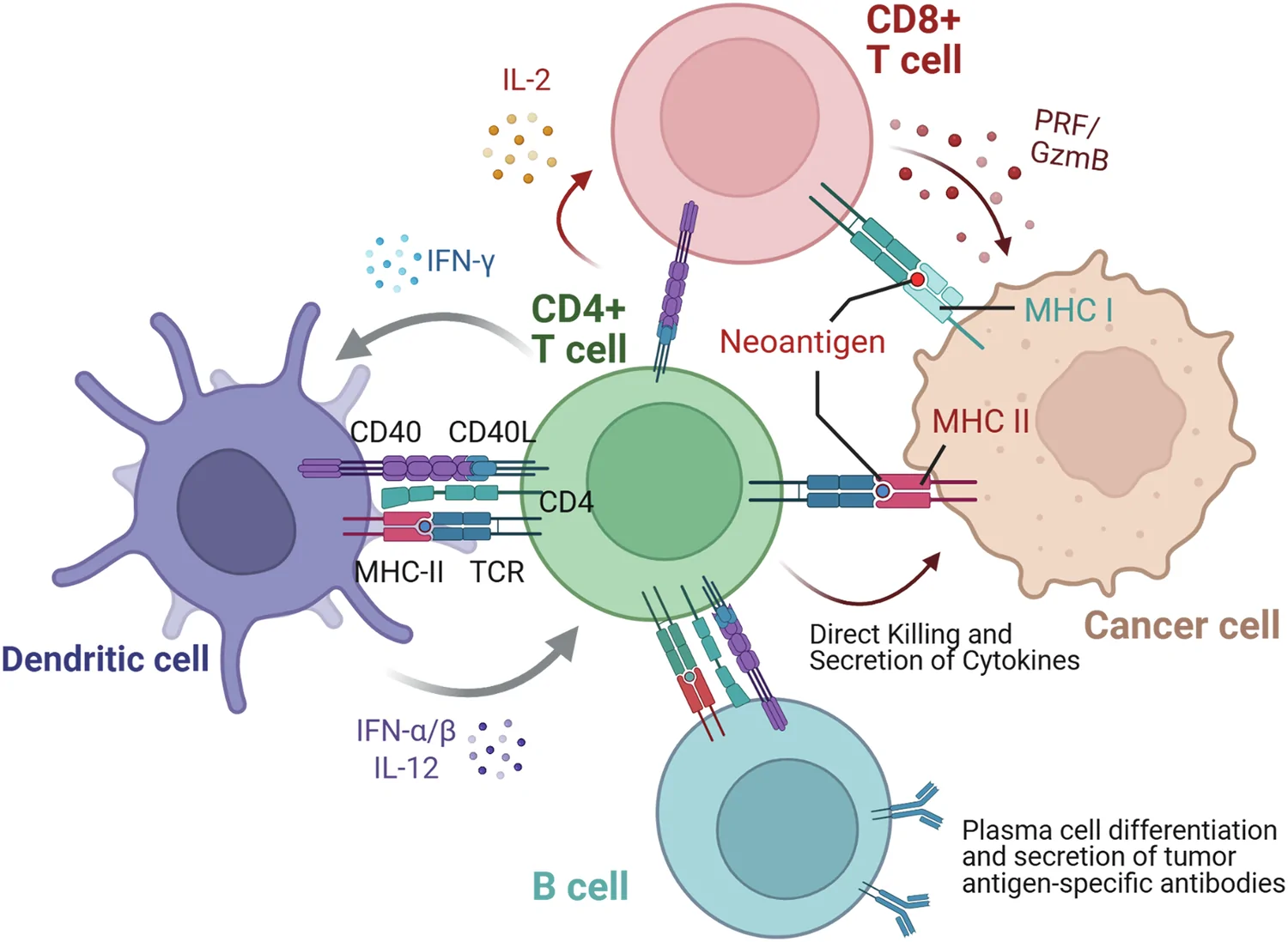
Multifunctional roles of CD4 T cells in cancer immunotherapy. CD4^+^ T cells have key roles in cancer immunotherapy. CD4^+^ T cells activate dendritic cells, prompting them to present antigens and boost the cytotoxic functions of CD8^+^ T cells. In addition, they activate B cells, which promote the secretion of tumor antigen-specific antibodies. IFN, interferon; MHC, major histocompatibility complex; PRF, perforin; TCR, T cell receptor.

CD4⁺ T cells complement and amplify these responses by providing essential “help” signals that support APCs and CD8⁺ T cell expansion^60–64^. T_H_1-differentiated CD4⁺ T cells, in particular, produce IL-2 and IFNγ, upregulate CD40L, and promote efficient cross-priming by DCs, thereby improving the generation of high-affinity CD8⁺ T cell responses (Fig. 3). In addition to their helper functions, a subset of cytotoxic CD4 T cells can directly kill MHC-II-expressing tumor cells, offering an additional therapeutic axis relevant to PCVs^65–67^. However, the CD4⁺ T cell compartment includes cell subsets such as T_H_2 cells and regulatory T cells, which can exert immunosuppressive effects. Moreover, immunosuppressive cells (MDSCs and M2-polarized macrophages) and increased expression of T cell exhaustion markers (PD-1, LAG-3, and TIM-3) can also weaken anticancer immune responses. In addition, tumor immune evasion mechanisms further limit the effectiveness of vaccine-induced T cell responses. For example, downregulation of HLA class I molecules or loss of heterozygosity at the HLA locus can impair antigen presentation, rendering neoantigen-bearing tumor cells less visible to CD8⁺ T cells. Intratumoral antigen heterogeneity also poses a major challenge, as subclonal neoantigens may be unevenly expressed across tumor cell populations, thereby reducing the effectiveness of vaccine-elicited T cells targeting a limited set of epitopes. Collectively, these mechanisms — together with T cell exhaustion and immunosuppressive TMEs — represent key biological barriers that must be overcome to achieve durable PCV efficacy. Therefore, these factors must be closely monitored in vaccine research^64,65^.

Collectively, these findings highlight that an optimal PCV design requires a balanced induction of both MHC-I-restricted CD8⁺ and MHC-II-restricted CD4⁺ responses. This dual-arm T cell activation provides an immunological foundation upon which downstream clinical efficacy depends and directly motivates the integration among antigen selection strategies, mRNA-based delivery platforms, and emerging clinical applications. Given that the magnitude, breadth, and persistence of vaccine-induced CD8⁺ and CD4⁺ T cell responses are strongly associated with clinical benefits, these mechanistic insights provide a conceptual basis for evaluating how effectively tailored cancer vaccines can reproduce these immune signatures in patients. This is crucial for confirming the clinical efficacy of PCV in real-world treatment settings.

## Clinical trials of personalized cancer vaccines

mRNA-based PCVs orchestrate multilayered adaptive immunity by mobilizing both CD8⁺ cytotoxic and CD4⁺ helper T cell networks. Given the evident translational relevance of these immune pathways, clinical trials testing whether such immunological signatures can be reproduced in patients and translated into meaningful therapeutic benefits are ongoing. PCVs have progressed from exploratory phase I studies to provide evidence of durable immune activation and early clinical benefits in several tumor types. Multiple vaccine modalities, including peptide-based, DC-based, viral vector-based, and mRNA-LNP-based platforms, have entered clinical investigations, with mRNA platforms advancing rapidly owing to their programmability and manufacturing agility^68^.

### Clinical experience with mRNA-based personalized vaccines

Among the various mRNA-based PCV programs currently under clinical investigation, a few landmark trials have generated the most mature evidence and now serve as the principal clinical reference points in the field. The studies highlighted below represent the most advanced and influential examples to date, illustrating how individualized mRNA vaccines perform across distinct tumor contexts.

#### mRNA-4157 (V940) in melanoma

Moderna’s individualized mRNA vaccine, mRNA-4157 (V940), encodes up to 34 patient-specific neoepitopes and has produced the most mature randomized data in the field. In the phase IIb KEYNOTE-942 trial, the combination of mRNA-4157 with pembrolizumab significantly reduced recurrence compared with pembrolizumab alone^69^. These findings are consistent with broader evidence that individualized neoantigen vaccination can augment checkpoint blockade. On the basis of these results, multiple phase III trials evaluating V940 expression in melanoma and non-small-cell lung cancer are currently ongoing.

#### mRNA vaccines in PDAC

Despite the low TMB and immunosuppressive TME of PDAC, personalized RNA vaccines have shown encouraging results. In a phase I clinical study, individualized neoantigen vaccines elicited durable CD8⁺ T cell responses in approximately half of the vaccinated patients^70^, with responders demonstrating delayed recurrence and prolonged RFS during long-term follow-up^71^. These outcomes are consistent with those of other early-phase trials that showed strong CD8⁺ priming in resected PDAC. Collectively, these findings support that early-stage PDAC, before extensive immunoediting, may be uniquely suited for effective PCV intervention.

Prominent clinical reports on patients with resected PDAC receiving individualized mRNA-LNP vaccines reinforce this idea by demonstrating the role of vaccine-primed CD8⁺ T cells in maintaining long-term remission in cancer types that are ordinarily associated with dismal outcomes. Notably, these cases involved tumors with low-to-intermediate TMB, indicating that a high mutational burden is not an absolute prerequisite for clinical benefit when high-quality clonal neoantigens are targeted early in the disease course. Together with formal trial data, these clinical observations have helped shift the perspective from viewing PCVs as a strategy reserved for TMB-high malignancies to regarding them as a platform that can unlock therapeutic vulnerabilities, even in low-TMB solid tumors such as PDAC.

### DC-based personalized vaccines

DC-based PCVs continued to demonstrate potent immunogenicity in early studies. In PDAC, autologous DCs pulsed with individualized neoantigen peptides induced CD4⁺ and CD8⁺ T cell responses in more than 80% of patients^72,73^. Several individuals maintained multiyear disease-free survival, highlighting the ability of ex vivo antigen loading approaches to generate robust immune activation, even in poorly immunogenic tumors. Provenge, the world’s first DC-based prostate cancer therapeutic vaccine, received FDA approval in 2010, with high market expectations; however, it failed commercially^74,75^ due to modest therapeutic efficacy, high production costs, and a complex manufacturing process. This DC-based cancer vaccine enhances the antigen-specific immune response against prostate cancer by administering APCs activated with PAP-granulocyte–macrophage colony-stimulating factor (GM-CSF), thereby augmenting that immune responses are insufficient when using wild-type APCs alone. However, its antitumor efficacy remains insufficient. Therefore, recent clinical trials are evaluating strategies that integrate mRNA technology with DCs or combine DC vaccination with immune checkpoint inhibitors (ICIs)^76,77^.

### Key insights from early trials

Across melanoma, PDAC, and other tumor types, the association between strong neoantigen-specific CD8⁺ T cell responses and improved clinical outcomes is evident^9,70,71^. This principle is reinforced by multiple mechanistic studies demonstrating the central role of vaccine-primed cytotoxic T cells in mediating tumor control^69,71^. Another consistent insight is that multi-epitope vaccine designs encoding diverse patient-specific neoantigens may help overcome intratumoral heterogeneity^9,23^. Finally, ongoing research suggests that vaccine-induced immunity may be further enhanced through immunomodulatory agents, such as IL-2, IL-12, IL-15, or GM-CSF^78^, which improve T cell survival, trafficking, and TME remodeling.

### Future clinical translation

PCV trials are increasingly incorporating earlier disease settings, serial immune monitoring, and antigen selection based on real-world immunogenicity data^45,47^. Simultaneously, improvements in automated manufacturing and modular vaccine design continue to accelerate the feasibility of individualized mRNA vaccine production^40,44,79^. These advancements collectively lay the foundation for next-generation PCV development, along with the expansion of combination strategies involving immunomodulating cytokines^78^.

Collectively, early-phase clinical trials have shown that PCVs can induce potent, mutation-specific T cell responses and, in select settings, generate measurable clinical benefits. However, they have also revealed critical bottlenecks in neoantigen selection, manufacturing timelines, delivery efficiency, and regulatory pathways that must be addressed for widespread clinical adoption of PCVs. These emerging challenges have motivated the strategic examination of key components required to optimize PCV development, production, and implementation.

## Five strategies for the optimal development of PCVs

**Fig. 4:**
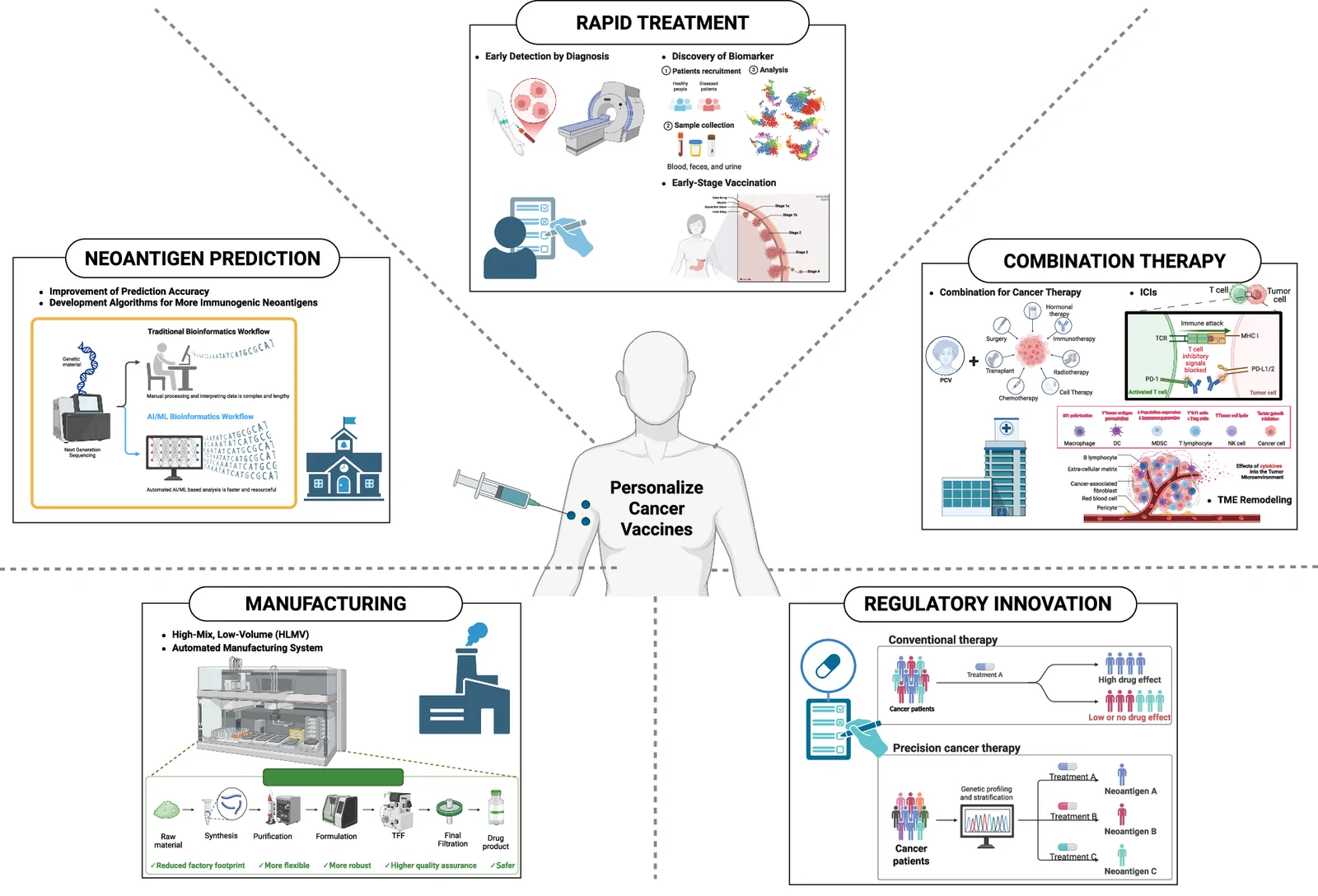
Five strategies for the development of PCVs. Neoantigen prediction requires not only improved antigen prediction accuracy but also advanced antigen prediction tools. For rapid treatment, early detection through frequent checkups is important. Combination therapeutic strategies and cost-effective manufacturing processes can lead to drugs that are more effective and accessible to patients. To achieve this innovation, the five strategies must be harmonized. AI, artificial intelligence; DC, dendritic cell; ICI, immune checkpoint inhibitor; MDSC, myeloid-derived suppressor cell; MHC, major histocompatibility complex; ML, machine learning; NK, natural killer; PCV, personalized cancer vaccine; TCR, T cell receptor; TME, tumor microenvironment; T_reg_, regulatory T.

Despite encouraging clinical signals, the widespread adoption of PCVs depends on overcoming several bottlenecks that span computational antigen discovery, patient selection, immunological optimization, manufacturing efficiency, and regulatory readiness. To address these challenges, we outline five strategic dimensions that collectively shape the translational trajectory of PCVs, from refining neoantigen prediction to accelerating production pipelines and establishing regulatory frameworks tailored to individualized therapies (Fig. 4).

### Improvement of neoantigen prediction techniques

Precise prediction of tumor antigens is a prerequisite for eliciting potent cancer-specific immune responses using PCVs. Recently, antigen prediction technology has evolved significantly alongside advanced bioinformatics, including next-generation sequencing, machine learning algorithms, and AI technologies. However, the current predictive accuracy remains suboptimal; recent studies have estimated that only ~6% of predicted neoantigens are genuinely immunogenic, highlighting the urgent need for methodological refinement^28^. In many cases, the algorithms used for neoantigen prediction are highly limited; in particular, most in silico predictions focus on MHC-I-specific neoantigens. This aligns with the general theory that MHC-I neoantigens typically exhibit anticancer effects^80^. However, animal and clinical research has demonstrated that MHC-II-specific neoantigens significantly influence anticancer efficacy^81–83^. Despite these advances, current prediction pipelines are associated with a high false-positive rate, with recent studies estimating that only ~6% of computationally predicted neoantigens are truly immunogenic. This discrepancy highlights a fundamental limitation in existing algorithms, which often prioritizes peptide–MHC binding affinity without sufficiently accounting for downstream processes required for effective antigen presentation and T cell activation. Several major hurdles impede the development of robust neoantigen prediction models, most notably tumor heterogeneity, HLA polymorphism, and the inherent limitations of existing algorithms and training datasets^84^. The heterogeneity of tumor tissues causes antigen profiles to vary depending on the sampling location, even within a single patient’s tumor tissue, leading to variations in antigen prediction. Moreover, HLA polymorphism complicates antigen prediction. Algorithms trained on limited datasets, including those lacking essential genomic, transcriptomic, or proteomic information, can lead to inaccurate antigen prediction.

This drive for precision has also reshaped the interpretation of TMB in both research and clinical practice. Although a high TMB often correlates with a greater pool of candidate epitopes, prediction pipelines increasingly emphasize the identification of clonally dominant MHC-presented peptides that are visible to T cells in vivo. Furthermore, although these algorithms primarily rely on binding affinity for MHC-I molecules, as noted earlier, MHC-II molecules are also crucial. Immunogenicity depends on multiple factors beyond MHC binding, including antigen processing, proteasomal cleavage, TAP transport efficiency, and TCR recognition^85^. In addition, variability in TCR repertoire among patients and differences in antigen presentation efficiency across tumor types further contribute to the unpredictability of neoantigen immunogenicity. These limitations underscore the need for next-generation prediction models that integrate multidimensional features, including antigen processing, presentation context, and immune recognition. Therefore, advancements that integrate these parameters are essential. Consequently, algorithms are moving beyond simple mutation counting toward integrated models that jointly consider the TMB, clonality, HLA genotype, transcript abundance, and proteasomal processing. This evolution reflects a growing consensus that the “quality” of neoantigens and their presentation context is more critical for PCVs than the absolute “quantity” or number of mutations per megabase^12,84,86^.

To enhance the accuracy of antigen prediction, it is necessary to improve our understanding of cancer through characteristic analysis that considers various factors such as cancer type, individual patient differences, sampling site, and sampling timing^87^. It is also important to standardize the dataset used to train the prediction algorithms, along with clear sources and procedures. Additionally, Ely et al.^88^ recently reported pancreatic cancer-specific cryptic antigens derived from untranslated regions, suggesting the potential for novel antigen discovery approaches beyond conventional somatic mutations. Finally, the next generation of predictive tools must evolve beyond merely assessing MHC–peptide–TCR binding affinity. Future algorithms should aim to predict the functional quality of the immune response by specifically targeting factors associated with the cytotoxic potential of T cells activated by selected neoantigens.

### Rapid deployment of PCVs

Cancer is particularly dangerous owing to its rapid growth and metastasis. Therefore, early detection of cancer is as important as the development of effective anticancer drugs, an importance that is even more pronounced in cancer immunotherapy^89^. In this context, the timing of vaccination is critical for anticancer efficacy, as the immune response induced by the vaccine can effectively halt cancer progression in the early stages^90^. In a recent clinical study of PCVs, Sethna et al.^71^ reported promising anticancer efficacy, including prolonged RFS, by priming long-lived CD8^+^ T cells following administration of RNA neoantigen vaccines to patients with pancreatic cancer. Interestingly, this study showed more promising results than previous PCV clinical trials. Notably, the patients were recruited based on cancer stage; patients with early-stage cancer, such as those with surgically resected margins classified as R0/R1, were selected, and those with metastatic, borderline, or locally unresectable PDAC were excluded. This suggests that PCV therapy is more effective in patients with early-stage cancer. The efficacy of promptly providing PCV therapy to patients in the early stages of cancer progression can be enhanced by the following: (1) rapid and accurate diagnosis through the discovery of advanced biomarkers^91,92^, (2) patient efforts through regular health check-ups, (3) manufacturers’ rapid production and supply of PCV, and (4) clinical trial design of PCVs targeting patients with early-stage cancer.

### Improvement of anticancer efficacy through combination therapy

Achieving anticancer efficacy by PCVs necessitates not only the induction of cancer-specific immune responses but also preventing the exhaustion of neoantigen-specific T cells, ensuring their infiltration into the TME, and maintenance of their activation within the TME^93^. Therefore, PCVs, similar to other anticancer therapies, should be considered in combination with drugs that can reduce tumor size and enhance T cell activation within the TME, rather than as a monotherapy. Anticancer therapies such as surgery, chemotherapy, radiotherapy, ICIs, adoptive cell therapy, cytokines, and small-molecule agents can achieve synergistic anticancer efficacy through combination therapy with PCVs^94,95^. Strategies used in clinical trials to induce synergistic anticancer effects through immunotherapy include maintaining T cells in an activated state using ICIs, enhancing cellular immune responses using pro-inflammatory cytokines, and adding immunostimulants capable of activating the innate immune system^68,96^.

ICIs are antibody therapies that enhance T cell anticancer efficacy by preventing T cell exhaustion. Well-known ICIs include PD-1 inhibitors, CTLA-4 inhibitors, and PD-L1 inhibitors, and efforts to discover effective and novel ICIs continue^97^. ICIs are already being studied as combination therapies in numerous global clinical trials for anticancer agents, and combination therapies with PCVs and ICIs are also being conducted in several clinical trials. For example, BioNTech confirmed the induction of immune responses by neoantigen-specific T cells when combined with anti-PD-L1 in a phase I clinical trial conducted in patients with PDAC and increased PDAC recurrence prevention and progression-free survival rates^70^. Moderna’s mRNA-4157 is an mRNA-based custom cancer vaccine that encodes 34 neoantigen sequences within the mRNA^69^. In clinical trials, when administered in combination with pembrolizumab (anti-PD-1) to patients with melanoma, it increased survival rates and reduced the risk of metastasis and death by ~65%.

Another combination therapy approach involves cytokines, which are biological molecules that have crucial roles in the proliferation, differentiation, and survival of immune cells^78,98^. Treatment with pro-inflammatory cytokines such as IFNγ, TNF-α, GM-CSF, IL-2, IL-12, and IL-15 has demonstrated preclinical or clinical relevance in cancer therapy^99^. Within the TME, they can suppress immune inhibitory cells, such as T_reg_ cells, tumor-associated macrophages, and MDSCs, induce cytotoxic T lymphocyte-mediated apoptosis, and remodel the TME^79^.

### Cost-effective manufacturing and rapid turnaround strategies

The manufacturing landscape of PCVs is inherently complex, as it necessitates the production of bespoke neoantigens with distinct sequences for each patient^29^. Unlike traditional biologics, each patient-specific batch requires rigorous quality control to ensure consistent performance independent of sequence variability. Consequently, conventional large-scale manufacturing models that rely on economies of scale are not suited for PCVs because of their prohibitive costs and lack of flexibility^89,90^. To address this economic bottleneck, it is imperative to establish a high-mix low-volume manufacturing ecosystem that is tailored to unique tumor profiles. However, the current scarcity of small-scale cGMP-compliant production facilities remains a critical limiting factor, increasing production costs and hindering widespread adoption^91^. In addition to cost, the time constraints of PCV production are critical. Currently, mRNA-based PCV production takes ~6–8 weeks from biopsy to patient administration^100^. This manufacturing time has been markedly reduced compared with the initial 8–10 weeks required, given the introduction of automated manufacturing processes and AI. As reducing this manufacturing time is considered one of the key factors in clinical success, both Moderna and BioNTech are intensively focussing their technological efforts on this area. Therefore, commercial viability hinges on the implementation of fully automated, modular systems capable of minimizing labor and overhead, alongside the development of innovative regulatory frameworks and sustained public–private investment.

In addition to the cost, the temporal constraints of PCV production are critical. Because the manufacturing workflow is triggered only after tissue procurement, rather than being off-the-shelf, minimizing the “vein-to-vein” turnaround time is essential for clinical efficacy. This requires a system design that integrates rapid delivery platforms, streamlined synthesis protocols, and accelerated release testing. However, the establishment of such an agile infrastructure is sensitive to macroeconomic conditions. The fluctuating public investment in mRNA research and recent budget contractions in major funding agencies pose significant risks. Without robust national support and the development of innovative reimbursement models, the technical feasibility of individualized vaccines may outpace their real-world accessibility^37,101^.

### Regulatory innovation

From a regulatory science perspective, PCVs present several distinct challenges compared with conventional pharmaceuticals, including the following: (1) validation of the antigen selection process, encompassing tissue sampling, molecular analysis, and in silico prediction^102^; (2) management of vaccine lot variability arising from patient-specific antigen sequences; and (3) establishment of manufacturing and quality control strategies capable of achieving current Good Manufacturing Practice compliance in small-batch production settings. Validation of the neoantigen selection process requires not only predicting antigens with high immunogenicity but also the development of reproducible, robust, and well-controlled workflows^103^. Validation of computational prediction processes is challenging because of the diversity of algorithms and statistical analysis tools used by different manufacturers, necessitating ongoing discussions with regulatory agencies. In pharmaceutical manufacturing, lot-to-lot variation owing to antigen sequence changes in PCVs is the most significant challenge and requires sufficient scientific evidence and validation to convince regulatory authorities. In addition, the supply of PCV for a single patient requires a small-batch manufacturing process to achieve efficient production costs. However, equipment suppliers and manufacturers tend to hesitate in building facilities for small-batch manufacturing processes that meet cGMP compliance, citing inefficient manufacturing costs and traditional pharmaceutical regulatory validation requirements. For example, traditional regulatory science and guidelines for bioburden monitoring and sterility testing in injectable drug manufacturing processes are difficult to apply to small-scale production systems because of their large sample sizes and long analysis times^104^. Recently, regulatory authorities in several major countries have published advanced therapy medical product guidelines to safely and effectively serve novel concept medicines based on cutting-edge technologies, including adoptive cell therapy, gene therapy, and tissue-engineered medicine^105^. Moreover, in Europe, the EMA’s PRIority MEdicines scheme and the 2025 guideline for investigational Advanced Therapy Medicinal Products provide a supportive regulatory framework for the development of individualized therapies^106^. In the USA, PCVs may likewise be considered for expedited regulatory programs within the therapeutic cancer vaccine framework, although the applicability of the Regenerative Medicine Advanced Therapy designation should be assessed on a case-by-case basis depending on whether the product meets the criteria for a regenerative medicine therapy^107^. These efforts by regulatory authorities are expected to drive the advancement of cutting-edge therapeutic systems, including personalized medicine, by bridging the gap between safety and innovation in pharmaceutical regulatory science. Ultimately, meaningful regulatory innovation will require close collaboration among the private sector, government, and public institutions toward the shared goal of improving patient health.

## Conclusion

PCVs have evolved from theoretical concepts in precision oncology to clinically substantiated immunotherapies capable of eliciting durable mutation-specific T cell responses. The convergence of multi-omics sequencing, neoantigen discovery, and mRNA-LNP engineering has established a robust technological foundation that enables vaccines to be tailored to the molecular identity of each tumor. Early clinical data on melanoma and pancreatic cancer have demonstrated that when applied within the optimal therapeutic window, these individualized modalities can generate substantive immunological and clinical benefits.

Despite these milestones, broad clinical implementation has been impeded by biological and logistical barriers. Neoantigen prediction remains constrained by intratumoral heterogeneity and incomplete immunogenicity data. Accumulated clinical experience has clarified that bulk TMB serves merely as a probabilistic enrichment marker rather than a deterministic predictor of benefit. It is now evident that the quality of neoantigens capable of inducing cancer-specific immune response, clonal dominance within tumor antigens, and the timing of vaccination often outweighs the number of mutations. Furthermore, the immunosuppressive TME can attenuate well-primed T cell responses, while manufacturing timelines frequently struggle to outpace disease progression. Additionally, regulatory frameworks originally designed for uniform biologics require adaptation to accommodate patient-specific production and rapid analytical workflows.

Consequently, the future trajectory of PCVs depends on the synergistic optimization of five critical domains. Consequently, the future trajectory of PCVs depends on the synergistic optimization of five critical domains. To provide a clearer perspective on future priorities, these key directions can be summarized as follows:

1. High-fidelity antigen prediction: Accurate identification of highly immunogenic and clonally dominant neoantigens is essential to improve therapeutic efficacy, particularly beyond conventional TMB-based selection strategies.
2. Early-stage clinical deployment: Timely administration of PCVs, especially in minimal residual disease or early-stage settings, is critical to maximize the effectiveness of vaccine-induced immune responses.
3. Combinatorial immunomodulatory strategies: Integration with ICIs, cytokines, and other immunotherapies is necessary to overcome T cell exhaustion and enhance TME remodeling.
4. Efficient small-batch manufacturing systems: The establishment of scalable high-mix, low-volume production platforms is required to reduce turnaround time and enable broader clinical implementation.
5. Adaptive regulatory frameworks: Flexible and innovation-driven regulatory strategies are needed to accommodate patient-specific vaccine production and ensure timely clinical translation.

Collectively, these structured priorities highlight that the successful clinical translation of PCVs will depend not on a single technological breakthrough, but on the coordinated advancement of these interconnected domains.

Integrating these elements provides the conceptual harmony needed to transform individualized vaccines from technically impressive prototypes into scalable therapies that are suitable for routine practice. Future iterations will likely feature multifactorial innovations including AI-driven antigen discovery, multifunctional mRNA constructs, and real-time quality assessment models. As these technologies converge, the unique mutational landscape of each tumor may be fully leveraged as a therapeutic vulnerability.

Scientific innovation must, ultimately, be matched through structural reforms. The current public discourse highlights the growing tension between the transformative potential of PCVs and the reality of constrained health-care resources. Achieving sustainable and equitable access requires cost structures compatible with individualized manufacturing, along with deliberate policy decisions and sustained investment. Addressing this gap is essential to ensure that the ability to harmonize the aforementioned five dimensions does not remain a privilege confined to select academic centers but translates into a broadly accessible treatment option for patients worldwide.
